# Reviving Intracranial Angioplasty and Stenting “SAMMPRIS and beyond”

**DOI:** 10.3389/fneur.2014.00101

**Published:** 2014-06-23

**Authors:** Muhammad U. Farooq, Firas Al-Ali, Jiangyong Min, Philip B. Gorelick

**Affiliations:** ^1^Division of Stroke and Vascular Neurology, Mercy Health Hauenstein Neurosciences at Saint Mary’s, Grand Rapids, MI, USA; ^2^Center for Neuro and Spine, Akron General Medical Center, Akron, OH, USA; ^3^Department of Translational Science and Molecular Medicine, Michigan State University College of Human Medicine, Grand Rapids, MI, USA

**Keywords:** intracranial stenosis, best medical therapy, neurointervention, angioplasty, stenting, Wingspan stent

## Abstract

We review the methods and results of Stenting and Aggressive Medical Management for Preventing Recurrent Stroke (SAMMPRIS) and provide a critical review of its strengths and limitations. In SAMMPRIS, the aggressive medical treatment arm (AMT arm) did substantially better than the Wingspan Stenting plus aggressive medical management arm (WS+ arm). Complications in the first 30 days post intervention led to the disparity between treatment arms. A major contribution of SAMMPRIS was the added value that AMT and lifestyle change may provide, when compared to a precursor trial, Warfarin–Aspirin Symptomatic Intracranial Disease (WASID), designed to prevent stroke in persons with high-grade symptomatic intracranial occlusive disease, however, the results of neither of these two trials have ever been reproduced. On the other hand, we argue that technical limitations of the Wingspan stent system (WS System) and lack of an angioplasty only intervention arm may have led to a premature launch of the trial and early termination of the study. Future randomized trials with different devices and modified patient selection criteria are warranted.

## Introduction

Recently, an important intracranial stenting prevention trial in patients with symptomatic intracranial atherosclerotic occlusive disease (sICAD), Stenting and Aggressive Medical Management for Preventing Recurrent Stroke (SAMMPRIS), was published (1). SAMMPRIS showed that AMT alone was superior to the Wingspan system plus aggressive medical therapy (WS+ arm). The main findings were unexpected by some. The publication of the results, we believe, has reduced intracranial endovascular revascularization (IER) therapies leaving those patients with intracranial atherosclerotic stenosis who have failed medical management without an alternative treatment strategy despite a high risk of stroke, minimum 12.2%, in the first year. In this topical review, we discuss the main results and limitation of SAMMPRIS, and re-address the question as to whether or not the findings were really surprising based on prior scientific information. In addition, we discuss strategies to advance the field of IER.

## Brief History of Carotid-Artery Surgery and Endovascular Interventions for Stroke Prevention: Lessons Learned

Carotid-artery reconstructive surgery for aneurysms and invasive local cancers was carried out as early as 1916 with resection and end-to-end anastomosis (2). By 1952, anastomotic techniques were well-described when substantial portions of the common and internal carotid arteries had to be sacrificed in the presence of local cancer. At this time, there was recognition of the importance of collateral circulation in conjunction with these types of anastomotic surgeries, as well as the importance of autogenous vein grafting (2). Whereas thrombosis of the common carotid artery had been described as early as 1881 and predilection for atherosclerosis at the carotid bifurcation and carotid siphon described in the 1900s, C. Miller Fisher’s report in 1951 has been considered the landmark article on this field (2). In this paper, a neuropathological correlation was emphasized. He argued for two stroke mechanisms: decreased flow by high-grade stenosis and embolic debris migrating downstream causing ischemic stroke. He also recognized the importance of collateral circulation in relation to permanency or occurrence of stroke symptoms and prophesized that surgical intervention might be possible (3).

Thromboendarterectomy was popularized in French literature in the 1940s (2), which consisted of resection of the intima and diseased media with the thrombus. However, it was not until the 1990s that carotid endarterectomy (CEA) was proven superior to medical management alone following several decades of surgical technique and instrumental refinements that also included a few failed trials that taught us how to improve our techniques and refine patient selection criteria (4, 5).

## Carotid Bifurcation Angioplasty and Stenting

Endovascular therapy for the cervical carotid-artery bifurcation with balloon angioplasty was reported in 1980 (6, 7) and it was shown to be safe and efficacious (8). Early experiences with balloon angioplasty, however, were complicated by the generation of embolic debris. Stenting was developed in response to the need for better outcomes after angioplasty and was proven to be effective by reducing the occurrence of plaque dislodgement, intimal dissection, elastic recoil of the vessel wall, and early and late stenosis (7).

The introduction of a protection device to catch the debris released during stenting, the basket, theoretically made the procedure safer and helped launch multiple studies comparing carotid-artery stenting (CAS) to CEA. Until recently, multiple trials comparing the efficacy and safety of endovascular stenting for carotid-artery bifurcation to CEA have been carried out with mixed results. The Carotid Revascularization Endarterectomy vs. Stenting Trial (CREST) demonstrated similar efficacy and safety outcomes between the two methods, but only after device improvement and refinement of patient selection (8–10). We suspect that the history of IER and stenting will experience similar challenges along the way until we establish the correct device, technique, and patient selection criteria.

## SAMMPRIS Methods and Study Design

Stenting and Aggressive Medical Management for Preventing Recurrent Stroke is a Phase III, investigator-initiated, multicenter, randomized, open label, stroke prevention trial funded by National Institute of Neurological Disorders and Stroke (NINDS) to determine whether the WS System angioplasty and stenting arm (WS+ arm) and intensive medical therapy are superior to intensive medical therapy alone (AMT arm) for preventing stroke in recently symptomatic patients with severe intracranial atherosclerotic stenosis. The trial was initiated in October 2008 and was conducted at 50 sites in the United States. The details of the study protocol have been described elsewhere (11).

Patients were randomized if they had TIA or non-disabling stroke within 30 days prior to enrollment attributed to 70–99% stenosis of a major intracranial artery. Randomization was at a 1:1 ratio to intensive medical therapy alone or to the WS+ arm.

### Primary end point

The primary endpoint of the trial was stroke or death within 30 days following enrollment or after a revascularization procedure for the qualifying event during the follow-up period, or stroke in the territory of the qualifying event beyond 30 days.

### Endovascular intervention

The Gateway angioplasty balloon (Boston Scientific, Fremont, CA, USA) and Wingspan stent (Boston Scientific, San Leandro, CA, USA) were the only devices allowed in the WS arm of the SAMMPRIS trial. The WS System was the only stent in the SAMMPRIS trial because it was the only FDA-approved device for use at the time of study.

### Intensive medical therapy

Intensive medical therapy in both intervention arms of the study consisted of aspirin (325 mg/day) for the entire follow-up period, clopidogrel (75 mg/day for 90 days) after enrollment, and aggressive risk factor management primarily targeting blood pressure to less than 130/80 mm Hg and low-density lipoprotein-cholesterol (LDL-C) concentration to <70 mg/dL by administration of antihypertensive agents and rosuvastatin, respectively. A neurologist, study coordinator, and lifestyle coach closely monitored patients. Medication compliance was closely monitored by the study coordinator and included pill counts and monitoring of the patients if they were taking antiplatelet medications, statin therapy, and other medications.

Patients were examined at enrollment, 30 days, and then every 4 months following enrollment. If blood pressure was not within target range, adjustments in medical treatment were made and the patient returned in 30 days for a follow-up visit (1).

### Statistical analysis

Based on the Warfarin–Aspirin Symptomatic Intracranial Disease (WASID) study, the final projected rate of the primary endpoint in the medical management group was 24.7% at 2 years taking into account a 15% relative risk reduction based on the influence of aggressive medical management. It was then estimated that 382 patients would be needed in each treatment arm to have 80% power to show a relative reduction of 35% favoring the WS arm (1).

### SAMMPRIS results

The 30-day rate of stroke and death was 14.7% in the WS arm (12.5% non-fatal stroke, 2.2% fatal stroke) and 5.8% in the medical arm (5.3% non-fatal stroke, 0.4% non-stroke death, *p* = 0.002), which resulted in the study being stopped prematurely. There were five stroke-related deaths in the WS arm and one non-stroke-related death in the medical arm within 30 days following enrollment. The 30-day rate of primary endpoint in the WS arm was higher than what the study investigators had anticipated (5.2–9.6%). Although there was no difference in main outcomes after 30 days of stroke (same territory, 13 patients in each arm), Kaplan–Meier curves were significantly different with 1-year rates of the primary endpoint between the WS arm (20.0%) and medical arm (12.2%, *p* = 0.009). When the study was stopped, 451 (59%) of the planned 764 patients had been enrolled; 227 were randomized to the treatment medical arm, and 224 were randomized to the WS arm. A futility analysis showed that there was essentially no chance that the WS arm would be proven superior to medical therapy (1).

Of the 224 patients randomized to the WS arm who underwent stenting (*n* = 219) or angioplasty alone (*n* = 5), 13 had hemorrhagic strokes. Seven of the 13 were intraparenchymal bleeds (IPH), all remote from the stented vessels. A subgroup analysis of the IPH showed its association with higher degrees of intracranial stenosis, administration of a preoperative clopidogrel loading dose of 600 mg, and high procedural activated clotting time of >300 s. Amongst the other hemorrhagic strokes, a total of four cases were subarachnoid hemorrhages (SAH).

## Discussion

### SAMMPRIS AMT arm and prior medical literature

Warfarin–Aspirin Symptomatic Intracranial Disease (WASID) demonstrated that subsequent stroke risk in patients with sICAD was related to the degree of vascular stenosis and the clinical presentation. A subsequent stroke risk in those patients was much higher than previously reported in other trials. In the WASID population, patients with >70% stenosis and TIA had a stroke rate in the first year equal to 14%, and 22.5% if they presented with stroke and for patients who presented with TIA or stroke and >70% stenosis, the combined stroke rate was 18% (12). Surprisingly in the SAMMPRIS AMT arm, the stroke rate was 12.2% in the first year, much lower than the results reported in WASID. Therefore, based on the above information, there are two possible explanations for the discrepancy with the WASID results. Either the WASID data exaggerated the true risk of symptomatic ICAD and SAMMPRIS results came to highlight this fact, or the WASID data were not generalizable to the SAMMPRIS patients.

In SAMMPRIS, however, AMT was applied to both the WS+ and AMT arms. Therefore, if aggressive medical therapy were to explain the difference between the results in the two treatment arms (WS+ arm vs. AMT arm), the effect of medication would have to differ between these two groups, favoring the AMT arm. We do not have a complete understanding of the profile and effect of medical risk factor control in the two treatment arms as long-term follow-up of study patients is currently underway. For there to be a differential effect in one treatment arm, control of key risks (e.g., glycosylated hemoglobin, hypertension, lipids, and physical exercise) would have to differ between the two arms thereby placing the WS+ arm at a disadvantage. Thus far, we have seen baseline and 4-month data in relation to key medical factors and the following observations have been made in the medical arm vs. WS+ arm at 4 months: systolic/diastolic blood pressure (134.8/77.3 vs. 133.1/76.2 mm Hg); LDL cholesterol (72.8 vs. 75.9 mg/dL); HDL cholesterol (41.9 vs. 43.2 mg/dL); non-HDL cholesterol (90.0 vs. 94.3 mg/dL); glycosylated hemoglobin (7.5 vs. 7.8%); current smoking (20.4 vs. 17.3%); moderate or vigorous exercise (56.6 vs. 56.1%). Thus, some of these factors slightly favor one treatment arm. Additional analyses and follow-up time will be required to determine the possible influence that these factors may have on the study outcomes. We are skeptical that these modest risk factor control differences between the intervention arms will have major influence on the primary study outcome.

One may consider the effect of combination therapy with aspirin plus clopidogrel on the results of SAMMPRIS. Since combination antiplatelet therapy was administered to patients in both treatment groups for the same period of time in this trial, the expected effect should be constant in both groups unless there was a differential negative effect, for example, in the WS+ arm, which does not seem to be the case. Several other aspects of combination antiplatelet therapy are of interest for further discussion. First, such combination therapy benefited smokers but not non-smokers in a non-primary analysis of the SAMMPRIS data. This may be an effect of more efficient conversion of the pro-drug clopidogrel to its active form by the 450 cytochrome system and has been observed in other studies (9, 13). Second, the rate of recurrent stroke in SAMMPRIS was about one-half that of the precursor study, WASID, which compared high-dose aspirin vs. warfarin (12.2 vs. 25%) (1, 14). However, if we exclude the perioperative strokes in SAMMPRIS then the rate of subsequent ischemic strokes in the territory of the qualifying artery was almost the same in the WS+ and medical arms. The 30-day rate of stroke or death in the angioplasty and stenting group was 14.7%, which is substantially higher than the rates previously reported ranging 4.4–9.6% (1). Therefore, we conclude that the SAMMPRIS medical regimen may be more advantageous than the WASID medical treatment regimen, and more careful control of vascular risk factors in SAMMPRIS was associated with lower risk of subsequent stroke (1, 14).

On the other hand, before the publication of the WASID study, the stroke rate in patients with intracranial atherosclerotic disease was on the order of 10–12% per year in multiple other studies (15, 16). Contrary to this data, WASID reported a much higher stroke rate (18%) per year for the patients with 70–99% stenosis (17). Although we do not know the precise degree of stenosis in the prior study, it is curious that the medical arm in SAMMPRIS found the same 12.2% rate of stroke, and we doubt the majority of cases in the prior study had <70% stenosis. As previously discussed, we wonder whether the WASID results were not generalizable to the SAMMPRIS study patients and thus, overestimated the real risk of subsequent stroke per year in patients with symptomatic ICAD in SAMMPRIS. However, one should interpret the findings with caution since neither the results of WASID nor those of SAMMPRIS have been reproduced in other studies as of yet. Therefore, at the present time such comparisons may not be valid, and their results still need to be validated by subsequent study. However, we believe that the risk of stroke in the first year in the vascular territory of symptomatic ICAD is at least 12% with best available AMT.

## Complications rate of the WS stent prior to launching SAMMPRIS

At a 14.7% complication rate within the first 30 days, the SAMMPRIS WS+ arm procedural complication rate was higher than anticipated. It was almost 2.5 times higher than that observed for stenting of symptomatic extracranial carotid-artery stenosis in CREST (8–10). The actual periprocedural complication rate was in the range of approximately 5–10 absolute percentage points higher than anticipated. However, we believe that the literature prior to the SAMMPRIS trial launch anticipated the actual complication rate. An early paper dealing with the complication rate of the WS System reported a 6.1% major periprocedural neurological complication rate (18). The important modifier “major” needs interpretation as all operators know that major complications are always less frequent than minor complications, triggering an expectation of an at least a 15% total complication rate (assuming major complications represent approximately 40% of all complications). This point was further validated in other studies (Al-Ali et al., May 2008, International Intracranial stenting conference. Ankara. Turkey), reporting their periprocedural complication rates at 3.6% major, 10.9% minor (total of 14.5%), and at 19.5% stroke/TIA rate at 1 year. In August of the same year, The NIH Multicenter Wingspan, Intracranial Stent, Registry Study results were reported and despite being retrospective and self-reported the stroke rate was at 14% at 6 months (19). Thus, concurrent available data on complication and outcome rates of stenting were generally higher than projected in SAMMPRIS and suggest the need for a different set of statistical calculations for the SAMMPRIS trial to avoid failure of the WS+ arm.

## Improving the Design of Subsequent Trials

A trial to test the merit of IER for stroke prevention in patients with symptomatic ICAD was, and is still needed. In this text, we have previously articulated certain reservations about the SAMMPRIS trial design, such as use of the WS system as the sole device allowed in the trial, despite the high complication rate previously reported in the literature and highlighted above. In addition, other reservations about SAMMPRIS include:

### Patient selection

There has been debate about whether high enough risk patients were enrolled in the study. Based on the WASID findings, we understand that those patients with 70–99% stenosis and TIA or stroke within 30 days before enrollment had the highest rate of ischemic stroke in the territory of the symptomatic artery (14). The WASID risk of TIA or stroke was 22.9% at 1 year and 25.0% at 2 years (14). SAMMPRIS was designed using risk estimates from this subgroup of the WASID trial. We agree that based on the WASID trial, the aforementioned patient risk profile was a reasonable one for choosing patients for eligibility in SAMMPRIS (11).

### Lesion morphology

The “Mori classification” [type A <5 mm in length, concentric or moderately eccentric, smooth stenosis; type B, 5–10 mm in length, extremely eccentric, or angulated (>45°), or irregular stenosis, or total occlusion (<3 months old); type C, >10 mm in length, extremely angulated (>90°) stenosis, or total occlusion (>3 months old), or lesion with a number of neovasculatures all around] was not clearly elucidated in the study design eligibility criteria, despite the fact that it has been well-documented in the literature (20). It has been shown that lesion length and morphology correlate with outcome following IER (20–22). For example, the intrastent multicenter registry showed much lower rates of neurological complications in patients with lesions <5 vs. 5- to 10-mm lesions or >10 mm lesions (23). Zhu et al. found a 12% rate of in-stent restenosis in Mori A lesions and a 50% rate in Mori C lesions (24). Another recent multicenter report of 670 treated lesions showed Mori A lesions were safer to treat and were less likely to develop restenosis (25). The lesions treated in the SAMMPRIS trial were either 14 mm in length or less (11, 26) but there was no stratification of the lesions along Mori or other system criteria to select for favorable lesions to treat.

### Fragile plaque and collateral circulation status

The presence of numerous micro embolic signals (MES) on Doppler ultrasound was found to predict a higher risk of subsequent stroke (27). Also, the WASID study revealed that patients with poor collateral circulation distal to the stenosis had higher risk of subsequent stroke. The SAMMPRIS trial did not include criteria taking into account MES or collateral circulation status. The impact of these factors on such a trial is not clear but needs to be further defined.

### Technique

Proper angioplasty technique “slow submaximal balloon inflation” was described in the late 1990s (28). The authors reported their experience and noticed that when they started using a smaller balloon 0.5 mm less than the diameter on the diseased vessel at its normal section and inflating it slowly over a period of 3–5 min to achieve nominal pressure, their complication rate dropped dramatically. The authors attributed this lower complication rate to decrease in frequency of large dissections at the angioplasty site. The findings were later confirmed in a major case series demonstrating that large dissection following angioplasty was associated with a statistically significant occurrence of stroke in the periprocedural period, and restenosis at follow-up (29). In SAMMPRIS, operators were encouraged to down size the balloon angioplasty by 0.5 mm, but this was not a requirement, nor was the slow inflation axiom. Since these data are not documented and not every patient had an angiography study following angioplasty and prior to stent placement, it is impossible to know with certainty the impact of the technique on the final trial results.

## Lessons Learned from SAMMPRIS Trial

We discuss below further insights from and since the publication of SAMMPRIS in relation to possible means to heighten the success of IER:

### Vessel size

Stenting and Aggressive Medical Management for Preventing Recurrent Stroke included vessels that were 2–4.5 mm in diameter. Vessel diameter was not a predictor of outcome.

### Vessels with perforators vs. vessels with no perforators

Stenting and Aggressive Medical Management for Preventing Recurrent Stroke demonstrated a higher risk of ischemic stroke during intervention in vessels with perforators (PV) than in those with no perforating vessels (nPV). For example, IER to the basilar artery had a higher complication rate than any other vessel. The importance of this distinction between PV vs. nPV has been confirmed and in direct comparison of outcomes following IER, it was found that different vessels carry a very different risk following IER. Vessels with perforators carried significantly higher risk following IER (MCA 16.3%, basilar artery 20.3%) than when there were nPV (vertebral artery 8.3%, internal carotid artery 4.9%) (29). Future trials should take this important information into consideration, by either avoiding PV until newer generation devices emerge, or by restricting intervention in some patients to balloon angioplasty using a significantly smaller diameter balloon and a shorter one.

### Role of operator and site experience

It is important to determine if the higher than expected rate of endovascular complications in the SAMMPRIS trial was related to the operator or site experience. The SAMMPRIS analysis showed that neurointerventionalists with less Wingspan experience did not have a higher rate of periprocedural strokes in the trial. Neurointerventionalists with a more than a 10-Wingspan case experience actually had higher rates of 30-day events than those with less than a 10-case experience (19.0 vs. 9.9%, *p* = 0.11). Moreover, high enrolling study sites in this trial had lower rates of hemorrhagic stroke; 9.8% at sites enrolling <12 patients vs. 2.7% at sites enrolling ≥12 patients (*p* = 0.04). The exact cause of this difference is not clear but most likely is related to factors other than the operators’ expertise, such as poor blood pressure control after stenting and reperfusion injury (30). Final review of SAMMPRIS results found no association between the operators’ exact prior experience and the outcome. Other authors have looked at the importance of the “learning curve” using the WS system (29). In their series, they observed that complications did not cluster at the beginning of their use of the WS system but rather, occurred along the whole period of their registry experience. This observation suggests that the notion of “increased familiarity with the stent or more selective choice of the operators would have altered the final results of the SAMMPRIS” is probably inaccurate.

## Challenges of the WS Stent

### The Wingspan stent

First, the WS stent most likely contributed to the complication rate in SAMMPRIS. The WS has numerous shortcomings including the need for an exchange length micro-wire that must be kept in place while exchanging the balloon catheter to the stent delivery catheter. This invariably causes back and forward motion of the micro-wire tip and possible vessel perforation. Second, the pusher used to stabilize and help deploy the stent that was very rigid and invariably causes tension and motion on the wire tip causing it sometimes to abruptly jump. Third, the stent delivery catheter is bulky (3.5 French) and advancing such a bulky catheter through the fresh angioplasty site would, at least theoretically, cause further injury to the blood vessel wall. Thus, a smaller delivery catheter is needed. Lastly, the opposition of the stent at the angioplasty site is suboptimal due to its lower WS stent radial force as compared to the balloon-mounted stent. This suboptimal stent opposition to the vessel wall can allow the persistence of tiny spaces between the stent strut and the vessel wall allowing for platelet aggregation. This may help explain the curious phenomenon seen with the use of WS stent, which is the occurrence of small strokes, days following the intervention. It is not always in the immediate aftermath of stent placement as it is customary when using the balloon angioplasty catheter or the balloon-mounted stent where delayed stroke almost always equates to stent thrombosis.

## Considerations for Future Trials

From the aforementioned information, we believe that we should now be able to improve the design of future IER trials based on better imaging techniques, patient and lesion selection, and improved procedural techniques. We make the following summary recommendations:

### Imaging

#### Digital conventional angiography

##### Degree of stenosis

Over the last several years, many reports have demonstrated that lesions more than 70% stenosis have higher risk of future stroke or TIA. Therefore, we can restrict our lesion selection to above 70% stenosis.

##### Lesion morphology

It has been shown repeatedly that Mori C lesions have a very high complication rate; hence, we believe these lesions should be excluded from intervention. Numerous reports have confirmed that lesions in the perforator vessels such as in the basilar or middle cerebral arteries have much higher complication rates than those in non-perforator vessels, and it could be that lesions in the perforator artery presenting with perforant territory stroke are riskier than those presenting in the perforator artery with distant stroke (31, 32). This point needs to be clarified before embarking on a new trial, as we mentioned above. We recommend a change in the device selection by restricting intervention in these lesions to angioplasty using balloon with smaller diameter and shorter length.

#### Magnetic resonance imaging

Magnetic resonance perfusion imaging is capable of demonstrating the patient with a focal area of relatively lower perfusion, indicating less robust collaterals. In the WASID study, these patients were shown to have a higher likelihood of subsequent stroke. Any future trial should consider including equal numbers of these patients in both treatment arms to decrease their potential-cofounding effects.

#### Doppler ultrasound

Since increase in number of MES correlates with increased chances of further stroke, taking this finding into account may help refine the selection of patients and lesions.

### Technical factors

Stenting and Aggressive Medical Management for Preventing Recurrent Stroke demonstrated that most of the complications were periprocedural ones. Hence, working hard to decrease these complications should impact any future trials in a positive way. We believe the following points are valid based on personal experience and review of the literature:

#### Guiding catheter positioning

It should be as close to the lesion as safely possible; intracranial internal carotid artery, or at C1/C2 level for the vertebral artery. Our rule of thumb “never more than four curves between the tip of the guiding catheter and the lesion.” This will decrease the jerky movement of the micro-wire tip during crossing the lesion and during any exchange of the micro-catheter system if it becomes needed. We believe that this requirement is so important that failure to place the guiding catheter in an acceptable position should be considered an exclusion criterion.

#### Angioplasty

Intracranial angioplasty can be performed relatively safely in most of the patients with intracranial stenosis. It appears that angioplasty has a much lower complication rate than any available stent on the market today. We believe that angioplasty should be the first line of intervention. Should it be attempted, we believe it should follow the axiom of submaximal, slow inflation technique. Currently available stents should be used only as a bail out for large dissection or significant recoiling of the lesion following angioplasty (29, 33, 34).

### Improving the available stent designs

Safer, more sophisticated stents are needed to improve outcomes of stenting procedures.

## Future Directions

The Wingspan self-expanding device used in the SAMMPRIS trial has potential technical drawbacks, and trials with newer stents and an angioplasty only arm are warranted. Overtime, more effective and safer endovascular procedures may be developed and further trials will be needed to determine if these procedures with advanced technology lower the risk of stroke compared with aggressive medical therapy in high-risk subgroups. Until the next stent generation emerges, angioplasty alone might be an option in some of the patients with intracranial stenosis and recurrent stroke after failure of best medical therapy. Moreover, several subgroups of patients with intracranial stenosis are at high risk of recurrent TIAs and strokes in spite of being on a best medical therapy such as those with posterior circulation involvement and high-grade stenosis, and others with recurrent ischemic events especially with blood pressure fluctuations (35). These patients may need neurointerventional procedures during the course of their intracranial stenosis management in spite of being on a best medical therapy due to recurrent ischemic events. Therefore, it is important to identify subgroups of patients who are at high risk of stroke despite being on an aggressive medical therapy protocol. However, it can be challenging as any neurointerventional procedure that aims to improve this outcome must have a low periprocedural complication rate and be able to lower the stroke rate over time when compared with the best medical therapy. The SAMMPRIS trial results encourage further research to investigate and find innovative ways of using endovascular therapies to treat severe symptomatic intracranial stenosis patients.

## Conflict of Interest Statement

Dr. Gorelick serves as a co-director of the Clinical Coordinating Center for the US DIAS 4 Trial sponsored by Lundbeck and his employer. Advantage Health – Saint Mary’s Medical Group has received honoraria from Vindico Medical Education for Dr. Gorelick’s participation in the development of acute stroke treatment educational programs. The other co-authors report no conflicts of interest.
